# DOES THE ASSOCIATION BETWEEN AGE AND MAJOR ILLNESS VARY BY NATIONAL HEALTHCARE QUALITY?

**DOI:** 10.1093/geroni/igz038.507

**Published:** 2019-11-08

**Authors:** Matthew A Andersson, Lindsay R Wilkinson, Markus H Schafer

**Affiliations:** 1 Baylor University, Waco, Texas, United States; 2 University of Toronto, Toronto, Ontario, Canada

## Abstract

Though the risk of chronic disease and disability accelerates once adults are in their 60s, 70s, and 80s, researchers have long suspected that economic, social, and institutional variation — even among high-income Western nations — may powerfully influence the likelihood that people remain healthy at advanced ages. This study builds on comparative research into global aging, by offering a multiple-indicator test of whether national healthcare quality modifies the association between age and major illness. Recent individual-level data on morbidity among respondents aged 50 or older (16 countries; 2014 European Social Survey) are merged with nation-level healthcare indicators. Healthcare quality is assessed using a subjective, evaluation-based approach (based on the 2011 International Social Survey Programme) and an objective, attributable-mortality approach (2010 Healthcare Access and Quality, based on the Global Burden of Disease Study). Lagged nation-level economic and health indicators are controlled to help isolate healthcare effects. Multilevel logistic and linear regression models of any major health condition and morbidity reveal that while older individuals showed approximately a 10% reduction in probability of major illness when residing in countries with higher healthcare quality, associations between age and morbidity indices combining number and severity of illness showed greater modification by healthcare quality, with reductions around 18%. Results across subjective and objective approaches to healthcare quality are strikingly consistent. Taken together, results are suggestive of healthcare’s protective role in reducing age-related illness and disability. Future research should illuminate pathways by which healthcare quality may lead to differences in healthy aging among advanced nations.

